# 2,4-Dichloro-*N*-(4-chloro­phen­yl)benzene­sulfonamide

**DOI:** 10.1107/S1600536810010494

**Published:** 2010-03-24

**Authors:** P. G. Nirmala, B. Thimme Gowda, Sabine Foro, Hartmut Fuess

**Affiliations:** a Department of Chemistry, Mangalore University, Mangalagangotri 574 199, Mangalore, India; b Institute of Materials Science, Darmstadt University of Technology, Petersenstrasse 23, D-64287 Darmstadt, Germany

## Abstract

The mol­ecule of the title compound, C_12_H_8_Cl_3_NO_2_S, is twisted at the S atom, the C—SO_2_—NH—C torsion angle being 67.8 (2)°. The dihedral angle between the two benzene rings is 65.0 (1)°. The crystal structure features inversion dimers linked by pairs of N—H⋯O hydrogen bonds.

## Related literature

For the preparation of the title compound, see: Savitha & Gowda (2006 ▶). For our study of the effect of substituents on the structures of *N*-(ar­yl)aryl­sulfonamides, see: Gowda *et al.* (2009 ▶, 2010 ▶). For related structures, see: Gelbrich *et al.* (2007 ▶); Perlovich *et al.* (2006 ▶).
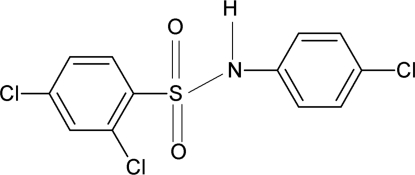


## Experimental

### 

#### Crystal data

C_12_H_8_Cl_3_NO_2_S
*M* *_r_* = 336.60
Triclinic, 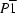

*a* = 6.3925 (9) Å
*b* = 10.524 (2) Å
*c* = 11.684 (2) Å
α = 69.51 (1)°
β = 77.96 (1)°
γ = 77.30 (1)°
*V* = 710.8 (2) Å^3^
*Z* = 2
Mo *K*α radiation
μ = 0.79 mm^−1^
*T* = 299 K
0.40 × 0.40 × 0.30 mm

#### Data collection

Oxford Diffraction Xcalibur single-crystal X-ray diffractometer with a Sapphire CCD detector
Absorption correction: multi-scan (*CrysAlis RED*; Oxford Diffraction, 2009 ▶) *T*_min_ = 0.744, *T*_max_ = 0.798
4462 measured reflections
2873 independent reflections
2514 reflections with *I* > 2σ(*I*)
*R*_int_ = 0.008

#### Refinement

*R*[*F* ^2^ > 2σ(*F* ^2^)] = 0.035
*wR*(*F* ^2^) = 0.091
*S* = 1.04
2873 reflections
175 parameters
1 restraint
H atoms treated by a mixture of independent and constrained refinement
Δρ_max_ = 0.41 e Å^−3^
Δρ_min_ = −0.47 e Å^−3^

### 

Data collection: *CrysAlis CCD* (Oxford Diffraction, 2009 ▶); cell refinement: *CrysAlis RED* (Oxford Diffraction, 2009 ▶); data reduction: *CrysAlis RED*; program(s) used to solve structure: *SHELXS97* (Sheldrick, 2008 ▶); program(s) used to refine structure: *SHELXL97* (Sheldrick, 2008 ▶); molecular graphics: *PLATON* (Spek, 2009 ▶); software used to prepare material for publication: *SHELXL97*.

## Supplementary Material

Crystal structure: contains datablocks I, global. DOI: 10.1107/S1600536810010494/ci5064sup1.cif

Structure factors: contains datablocks I. DOI: 10.1107/S1600536810010494/ci5064Isup2.hkl

Additional supplementary materials:  crystallographic information; 3D view; checkCIF report

## Figures and Tables

**Table 1:** Hydrogen-bond geometry (Å, °)

*D*—H⋯*A*	*D*—H	H⋯*A*	*D*⋯*A*	*D*—H⋯*A*
N1—H1*N*⋯O2^i^	0.84 (2)	2.20 (2)	3.014 (3)	163 (2)

Symmetry code: (i) 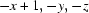.

**Fig. 1.**
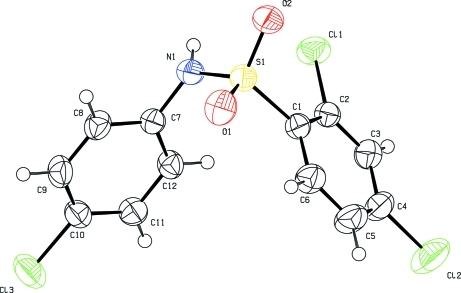
Molecular structure of (I), showing the atom-labelling scheme. Displacement ellipsoids are drawn at the 50% probability level.

**Fig. 2.**
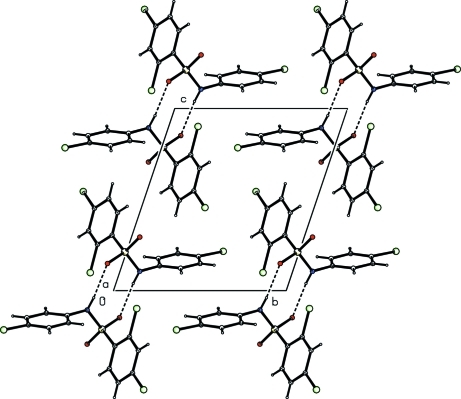
Molecular packing of (I) with hydrogen bonding shown as dashed lines.

## supplementary crystallographic information

### Comment

In the present work, as part of a study of substituent effects on the structures of *N*-(aryl)arylsulfonamides (Gowda *et al.*, 2009, 2010), the structure of 2,4-dichloro-*N*-(4-chlorophenyl)benzenesulfonamide (I) has been determined (Fig. 1). The molecule is twisted at the S—N bond with the C—SO_2_—NH—C torsion angle being 67.8 (2)° compared to the values of 60.6 (4)° (molecule 1), -59.7 (3)° (molecule 2), 63.9 (4)° (molecule 3) and 53.0 (4)° (molecule 4), in the four molecules of 2,4-dichloro-*N*- (4-methylphenyl)benzenesulfonamide (II) (Gowda *et al.*, 2010) and -48.2 (2)° in 2,4-dichloro-*N*-(3,4-dichlorophenyl)benzenesulfonamide (III) (Gowda *et al.*, 2009).

The sulfonyl benzene and the aniline benzene rings in (I) are tilted relative to each other by 65.0 (1)°, compared to the values of 85.2 (1)° (molecule 1), 80.5 (2)° (molecule 2, disordered orientation A), 80.1 (2)° (molecule 2, orientation B), 87.5 (7) (molecule 3, disordered orientation A), 87.0 (6)° (molecule 3, orienation B) and 72.4 (1)° (molecule 4) in the four molecules of (II) and 68.9 (1)° in (III). The other bond parameters in (I) are similar to those observed in (II), (III) and other aryl sulfonamides (Perlovich *et al.*, 2006; Gelbrich *et al.*, 2007).

In the crystal structure, the pairs of intermolecular N—H···O hydrogen bonds (Table 1) link the molecules to form inversion-related dimers as shown in Fig. 2.

### Experimental

The solution of 1,3-dichlorobenzene (10 ml) in chloroform (40 ml) was treated drop-wise with chlorosulfonic acid (25 ml) at 273 K. After the initial evolution of hydrogen chloride subsided, the reaction mixture was brought to room temperature and poured into crushed ice in a beaker. The chloroform layer was separated, washed with cold water and allowed to evaporate slowly. The residual 2,4-dichlorobenzenesulfonylchloride was treated with a stoichiometric amount of *p*-chloroaniline and boiled for ten minutes. The reaction mixture was then cooled to room temperature and added to ice cold water (100 ml). The resultant solid 2,4-dichloro-*N*- (4-chlorophenyl)benzenesulfonamide was filtered under suction and washed thoroughly with cold water. It was then recrystallized to constant melting point from dilute ethanol. The purity of the compound was checked and characterized by recording its infrared and NMR spectra (Savitha & Gowda, 2006). The single crystals used in X-ray diffraction studies were grown in ethanolic solution by slow evaporation at room temperature.

### Refinement

The H atom of the NH group was located in a difference map and refined with the distance restraint N–H = 0.86 (2) Å. The other H atoms were positioned with idealized geometry using a riding model [C–H = 0.93 Å]. All H atoms were refined with isotropic displacement parameters (set to 1.2 times of the *U*_eq_ of the parent atom).

### Crystal data

**Table:**

C_12_H_8_Cl_3_NO_2_S	*Z* = 2
*M**_r_* = 336.60	*F*(000) = 340
Triclinic, *P*1	*D*_x_ = 1.573 Mg m^−^^3^
Hall symbol: -P 1	Mo *K*α radiation, λ = 0.71073 Å
*a* = 6.3925 (9) Å	Cell parameters from 2802 reflections
*b* = 10.524 (2) Å	θ = 3.2–27.7°
*c* = 11.684 (2) Å	µ = 0.79 mm^−^^1^
α = 69.51 (1)°	*T* = 299 K
β = 77.96 (1)°	Prism, colourless
γ = 77.30 (1)°	0.40 × 0.40 × 0.30 mm
*V* = 710.8 (2) Å^3^	

### Data collection

**Table:**

Oxford Diffraction Xcalibur single-crystal X-ray diffractometer with a Sapphire CCD detector	2873 independent reflections
Radiation source: fine-focus sealed tube	2514 reflections with *I* > 2σ(*I*)
graphite	*R*_int_ = 0.008
Rotation method data acquisition using ω and φ scans	θ_max_ = 26.4°, θ_min_ = 3.2°
Absorption correction: multi-scan (*CrysAlis RED*; Oxford Diffraction, 2009)	*h* = −7→7
*T*_min_ = 0.744, *T*_max_ = 0.798	*k* = −13→13
4462 measured reflections	*l* = −14→12

### Refinement

**Table:**

Refinement on *F*^2^	Primary atom site location: structure-invariant direct methods
Least-squares matrix: full	Secondary atom site location: difference Fourier map
*R*[*F*^2^ > 2σ(*F*^2^)] = 0.035	Hydrogen site location: inferred from neighbouring sites
*wR*(*F*^2^) = 0.091	H atoms treated by a mixture of independent and constrained refinement
*S* = 1.04	*w* = 1/[σ^2^(*F*_o_^2^) + (0.0386*P*)^2^ + 0.4934*P*] where *P* = (*F*_o_^2^ + 2*F*_c_^2^)/3
2873 reflections	(Δ/σ)_max_ = 0.001
175 parameters	Δρ_max_ = 0.41 e Å^−^^3^
1 restraint	Δρ_min_ = −0.47 e Å^−^^3^

### Special details

**Table:**

Geometry. All esds (except the esd in the dihedral angle between two l.s. planes) are estimated using the full covariance matrix. The cell esds are taken into account individually in the estimation of esds in distances, angles and torsion angles; correlations between esds in cell parameters are only used when they are defined by crystal symmetry. An approximate (isotropic) treatment of cell esds is used for estimating esds involving l.s. planes.
Refinement. Refinement of *F*^2^ against ALL reflections. The weighted *R*-factor wR and goodness of fit *S* are based on *F*^2^, conventional *R*-factors *R* are based on *F*, with *F* set to zero for negative *F*^2^. The threshold expression of *F*^2^ > σ(*F*^2^) is used only for calculating *R*-factors(gt) etc. and is not relevant to the choice of reflections for refinement. *R*-factors based on *F*^2^ are statistically about twice as large as those based on *F*, and *R*- factors based on ALL data will be even larger.

### Fractional atomic coordinates and isotropic or equivalent isotropic displacement parameters (Å^2^)

**Table:**

	*x*	*y*	*z*	*U*_iso_*/*U*_eq_	
C1	0.2300 (3)	−0.1157 (2)	0.30386 (18)	0.0362 (4)	
C2	0.1233 (3)	−0.1828 (2)	0.25451 (18)	0.0370 (4)	
C3	−0.0339 (4)	−0.2602 (2)	0.3281 (2)	0.0449 (5)	
H3	−0.1049	−0.3044	0.2949	0.054*	
C4	−0.0835 (4)	−0.2707 (2)	0.4523 (2)	0.0504 (5)	
C5	0.0190 (4)	−0.2058 (3)	0.5036 (2)	0.0553 (6)	
H5	−0.0168	−0.2136	0.5869	0.066*	
C6	0.1766 (4)	−0.1286 (2)	0.4290 (2)	0.0477 (5)	
H6	0.2472	−0.0850	0.4629	0.057*	
C7	0.1442 (3)	0.2193 (2)	0.13604 (18)	0.0357 (4)	
C8	0.1996 (3)	0.3448 (2)	0.1231 (2)	0.0430 (5)	
H8	0.3429	0.3588	0.0971	0.052*	
C9	0.0426 (4)	0.4488 (2)	0.1487 (2)	0.0473 (5)	
H9	0.0792	0.5329	0.1397	0.057*	
C10	−0.1696 (3)	0.4259 (2)	0.1880 (2)	0.0421 (5)	
C11	−0.2275 (3)	0.3009 (2)	0.2035 (2)	0.0420 (5)	
H11	−0.3703	0.2865	0.2317	0.050*	
C12	−0.0694 (3)	0.1975 (2)	0.17658 (19)	0.0397 (4)	
H12	−0.1065	0.1135	0.1857	0.048*	
N1	0.3104 (3)	0.11422 (18)	0.10621 (17)	0.0432 (4)	
H1N	0.312 (4)	0.100 (3)	0.0395 (18)	0.052*	
O1	0.4832 (3)	0.04803 (18)	0.29394 (16)	0.0551 (4)	
O2	0.5937 (2)	−0.08756 (17)	0.14887 (15)	0.0529 (4)	
Cl1	0.17630 (10)	−0.16945 (7)	0.09860 (5)	0.05343 (17)	
Cl2	−0.28179 (14)	−0.36791 (9)	0.54410 (7)	0.0830 (3)	
Cl3	−0.36952 (11)	0.55863 (7)	0.21560 (8)	0.0699 (2)	
S1	0.42695 (8)	−0.00974 (5)	0.21258 (5)	0.03973 (14)	

### Atomic displacement parameters (Å^2^)

**Table:**

	*U*^11^	*U*^22^	*U*^33^	*U*^12^	*U*^13^	*U*^23^
C1	0.0351 (10)	0.0349 (10)	0.0358 (10)	−0.0039 (8)	−0.0043 (8)	−0.0094 (8)
C2	0.0393 (10)	0.0357 (10)	0.0334 (10)	−0.0018 (8)	−0.0043 (8)	−0.0109 (8)
C3	0.0463 (12)	0.0424 (11)	0.0464 (12)	−0.0104 (9)	−0.0050 (9)	−0.0138 (9)
C4	0.0497 (13)	0.0499 (13)	0.0429 (12)	−0.0139 (10)	0.0019 (10)	−0.0056 (10)
C5	0.0659 (15)	0.0634 (15)	0.0325 (11)	−0.0148 (12)	−0.0011 (10)	−0.0112 (10)
C6	0.0557 (13)	0.0513 (13)	0.0384 (11)	−0.0118 (10)	−0.0073 (10)	−0.0149 (10)
C7	0.0369 (10)	0.0344 (10)	0.0335 (9)	−0.0039 (8)	−0.0044 (8)	−0.0095 (8)
C8	0.0365 (10)	0.0412 (11)	0.0480 (12)	−0.0122 (9)	−0.0032 (9)	−0.0081 (9)
C9	0.0525 (13)	0.0337 (10)	0.0574 (13)	−0.0131 (9)	−0.0071 (10)	−0.0134 (9)
C10	0.0425 (11)	0.0373 (10)	0.0454 (11)	−0.0003 (9)	−0.0070 (9)	−0.0152 (9)
C11	0.0340 (10)	0.0450 (11)	0.0483 (12)	−0.0082 (9)	−0.0047 (9)	−0.0159 (9)
C12	0.0402 (10)	0.0365 (10)	0.0462 (11)	−0.0098 (8)	−0.0065 (9)	−0.0154 (9)
N1	0.0445 (10)	0.0402 (9)	0.0379 (9)	−0.0001 (8)	−0.0002 (8)	−0.0113 (8)
O1	0.0531 (9)	0.0575 (10)	0.0612 (10)	−0.0182 (8)	−0.0146 (8)	−0.0175 (8)
O2	0.0368 (8)	0.0504 (9)	0.0592 (10)	0.0036 (7)	0.0019 (7)	−0.0141 (8)
Cl1	0.0641 (4)	0.0632 (4)	0.0384 (3)	−0.0168 (3)	0.0000 (2)	−0.0230 (3)
Cl2	0.0816 (5)	0.0924 (6)	0.0669 (5)	−0.0470 (4)	0.0183 (4)	−0.0121 (4)
Cl3	0.0592 (4)	0.0491 (3)	0.0987 (5)	0.0051 (3)	0.0000 (4)	−0.0350 (4)
S1	0.0324 (3)	0.0389 (3)	0.0448 (3)	−0.0049 (2)	−0.0031 (2)	−0.0116 (2)

### Geometric parameters (Å, °)

**Table:**

C1—C6	1.395 (3)	C7—N1	1.440 (3)
C1—C2	1.398 (3)	C8—C9	1.384 (3)
C1—S1	1.787 (2)	C8—H8	0.93
C2—C3	1.387 (3)	C9—C10	1.384 (3)
C2—Cl1	1.743 (2)	C9—H9	0.93
C3—C4	1.389 (3)	C10—C11	1.384 (3)
C3—H3	0.93	C10—Cl3	1.746 (2)
C4—C5	1.380 (4)	C11—C12	1.389 (3)
C4—Cl2	1.743 (2)	C11—H11	0.93
C5—C6	1.391 (3)	C12—H12	0.93
C5—H5	0.93	N1—S1	1.6203 (19)
C6—H6	0.93	N1—H1N	0.839 (16)
C7—C12	1.391 (3)	O1—S1	1.4305 (17)
C7—C8	1.391 (3)	O2—S1	1.4398 (16)
			
C6—C1—C2	118.88 (19)	C7—C8—H8	119.8
C6—C1—S1	118.25 (16)	C10—C9—C8	119.11 (19)
C2—C1—S1	122.84 (15)	C10—C9—H9	120.4
C3—C2—C1	120.79 (18)	C8—C9—H9	120.4
C3—C2—Cl1	117.32 (16)	C9—C10—C11	121.48 (19)
C1—C2—Cl1	121.87 (15)	C9—C10—Cl3	119.21 (17)
C2—C3—C4	118.9 (2)	C11—C10—Cl3	119.30 (16)
C2—C3—H3	120.6	C10—C11—C12	119.08 (19)
C4—C3—H3	120.6	C10—C11—H11	120.5
C5—C4—C3	121.7 (2)	C12—C11—H11	120.5
C5—C4—Cl2	119.76 (18)	C11—C12—C7	120.12 (18)
C3—C4—Cl2	118.58 (19)	C11—C12—H12	119.9
C4—C5—C6	119.0 (2)	C7—C12—H12	119.9
C4—C5—H5	120.5	C7—N1—S1	121.38 (14)
C6—C5—H5	120.5	C7—N1—H1N	117.2 (18)
C5—C6—C1	120.8 (2)	S1—N1—H1N	117.4 (18)
C5—C6—H6	119.6	O1—S1—O2	119.68 (10)
C1—C6—H6	119.6	O1—S1—N1	108.21 (10)
C12—C7—C8	119.89 (18)	O2—S1—N1	105.97 (10)
C12—C7—N1	121.13 (18)	O1—S1—C1	105.62 (10)
C8—C7—N1	118.99 (18)	O2—S1—C1	109.05 (10)
C9—C8—C7	120.31 (19)	N1—S1—C1	107.86 (10)
C9—C8—H8	119.8		
			
C6—C1—C2—C3	0.4 (3)	C8—C9—C10—Cl3	−177.94 (18)
S1—C1—C2—C3	−177.75 (16)	C9—C10—C11—C12	−1.4 (3)
C6—C1—C2—Cl1	178.71 (16)	Cl3—C10—C11—C12	177.38 (17)
S1—C1—C2—Cl1	0.6 (2)	C10—C11—C12—C7	0.8 (3)
C1—C2—C3—C4	−0.3 (3)	C8—C7—C12—C11	0.3 (3)
Cl1—C2—C3—C4	−178.66 (17)	N1—C7—C12—C11	−179.30 (19)
C2—C3—C4—C5	0.2 (4)	C12—C7—N1—S1	−81.8 (2)
C2—C3—C4—Cl2	179.86 (17)	C8—C7—N1—S1	98.6 (2)
C3—C4—C5—C6	−0.3 (4)	C7—N1—S1—O1	−46.02 (19)
Cl2—C4—C5—C6	−179.93 (19)	C7—N1—S1—O2	−175.53 (16)
C4—C5—C6—C1	0.4 (4)	C7—N1—S1—C1	67.79 (18)
C2—C1—C6—C5	−0.5 (3)	C6—C1—S1—O1	−2.80 (19)
S1—C1—C6—C5	177.76 (19)	C2—C1—S1—O1	175.37 (16)
C12—C7—C8—C9	−0.9 (3)	C6—C1—S1—O2	127.02 (17)
N1—C7—C8—C9	178.7 (2)	C2—C1—S1—O2	−54.81 (19)
C7—C8—C9—C10	0.3 (3)	C6—C1—S1—N1	−118.33 (17)
C8—C9—C10—C11	0.8 (3)	C2—C1—S1—N1	59.84 (19)

### Hydrogen-bond geometry (Å, °)

**Table:**

*D*—H···*A*	*D*—H	H···*A*	*D*···*A*	*D*—H···*A*
N1—H1N···O2^i^	0.84 (2)	2.20 (2)	3.014 (3)	163 (2)

Symmetry codes: (i) −*x*+1, −*y*, −*z*.
